# The sequencing and *de novo* assembly of the *Larimichthys crocea* genome using PacBio and Hi-C technologies

**DOI:** 10.1038/s41597-019-0194-3

**Published:** 2019-10-01

**Authors:** Baohua Chen, Zhixiong Zhou, Qiaozhen Ke, Yidi Wu, Huaqiang Bai, Fei Pu, Peng Xu

**Affiliations:** 1 State Key Laboratory of Large Yellow Croaker Breeding, Ningde Fufa Fisheries Company Limited, Ningde, 352130 China; 2 State Key Laboratory of Marine Environmental Science, College of Ocean and Earth Sciences, Xiamen University, Xiamen, 361102 China; 3 Laboratory for Marine Biology and Biotechnology, Qingdao National Laboratory for Marine Science and Technology, Qingdao, 266071 China

**Keywords:** Sequencing, Genome, DNA sequencing, Ichthyology

## Abstract

*Larimichthys crocea* is an endemic marine fish in East Asia that belongs to Sciaenidae in *Perciformes*. *L*. *crocea* has now been recognized as an “iconic” marine fish species in China because not only is it a popular food fish in China, it is a representative victim of overfishing and still provides high value fish products supported by the modern large-scale mariculture industry. Here, we report a chromosome-level reference genome of *L*. *crocea* generated by employing the PacBio single molecule sequencing technique (SMRT) and high-throughput chromosome conformation capture (Hi-C) technologies. The genome sequences were assembled into 1,591 contigs with a total length of 723.86 Mb and a contig N50 length of 2.83 Mb. After chromosome-level scaffolding, 24 scaffolds were constructed with a total length of 668.67 Mb (92.48% of the total length). Genome annotation identified 23,657 protein-coding genes and 7262 ncRNAs. This highly accurate, chromosome-level reference genome of *L*. *crocea* provides an essential genome resource to support the development of genome-scale selective breeding and restocking strategies of *L*. *crocea*.

## Abstract (summary)

**Table:** 

Measurement(s)	reference genome data
Technology Type(s)	DNA sequencing
Sample Characteristic - Organism	Larimichthys crocea

Machine-accessible metadata file describing the reported data: 10.6084/m9.figshare.9767096

## Background & Summary

*Larimichthys crocea*, as known as large yellow croaker, is an endemic marine fish in East Asia that belongs to Sciaenidae in *Perciformes*. *L*. *crocea* has been ranked as one of the top commercial marine fishery species in China in the past two centuries. According to a Food and Agriculture Organization (FAO) estimate, the fraction of the world’s marine fish stocks fished at biologically unsustainable levels have reached 33.1% in 2015^1^, and among them, *L*. *crocea* has been widely recognized as one of the most depleted and threatened marine fishery species in China due to overfishing in the 1970s and 1980s^2^. A method of artificial reproduction/propagation for *L*. *crocea* was successfully developed based on a small group of wild *L*. *crocea* adults collected from the wild population in Fujian Province in the late 1980s. Since then, offshore mariculture of *L*. *crocea* has grown quickly in the past two decades, and it became the top mariculture fish in China with an annual production of 177,640 tons in 2017^3^.

*L*. *crocea* is now recognized as an “iconic” marine fish species in China because not only is it a popular food fish in China, it is a representative victim of overfishing and still provides high value fish products supported by the modern large-scale mariculture industry. Due to its impressive economic value in China and importance for marine biodiversity, abundant genome resources and genetic tools for this fish have been developed, including two genetic maps^4,5^, two draft genomes generated based on Illumina technology^6,7^ and a recently published draft genome using PacBio sequencing technology^8^ (which can be accessed via NCBI BioProject database, accession ID PRJNA480121). However, a chromosome-level, highly accurate reference genome is still lacking for *L*. *crocea* hindering genome-scale genetic breeding, conservation and restocking evaluation for sustainable aquaculture of *L*. *crocea*.

In this report, we provided chromosome-level reference genome sequences of *L*. *crocea* combining the PacBio single molecule sequencing technique (SMRT) and high-throughput chromosome conformation capture (Hi-C) technologies.

In addition, we also produced a chromosome-level reference genome of *Takifugu bimaculatus*^9^, which is also cultured as an important food fish in China, via almost the same approach. Both genomes were assembled with high quality, confirming the stability and suitability of this approach for marine fishes. The availability of a fully sequenced and annotated genome is essential to support basic genetic studies and will be helpful to develop genome-scale selective breeding strategies for these important mariculture species.

## Methods

### Sample collection, library construction and sequencing

A healthy female large yellow croaker belonging to the F1 generation of the “Fufa I” strain was collected from the State Key Laboratory of Large Yellow Croaker Breeding at Ningde, Fujian Province, China, and white muscle samples were collected. The muscle samples were immediately frozen in liquid nitrogen for 30 min and then stored at −80 °C. For high-molecular-weight (HMW) genomic DNA (gDNA) extraction, frozen samples were lysed in SDS digestion buffer with proteinase K. Then, the lysates were purified using AMPure XP beads (Beckman Coulter, High Wycombe, UK) to obtain HMW gDNA. Meanwhile, normal-molecular-weight (NMW) gDNA was extracted from the same samples using the DNeasy 96 Blood and Tissue Kit (Qiagen, Shanghai, China).

A whole-genome shotgun sequencing strategy was employed for genome size estimation and polishing of preliminary contigs. An Illumina library with 250 bp insert size was constructed from NMW gDNA using the standard protocol provided by Illumina (San Diego, CA, USA), and paired-end sequencing was performed using the Illumina HiSeq2500 platform with a read length of 2 × 150 bp. Finally, 105.23 Gb raw reads were generated. All reads containing adaptor sequences were discarded first. After that, uncertain bases (represented by “N”) and low-quality bases (Q < 5) were trimmed from the remaining Illumina reads using SolexaQA ++ ^10^ (version v.3.1.7.1). After trimming, there was a total of 105.01 Gb reads longer than 30 bp remaining, and these were retained as clean reads and used in genome size estimation and preliminary contig polishing (Table 1).

**Table 1:** Summary of obtained data using multiple sequencing technologies.

Library Type	Insert Size (bp)	Raw Data (Gb)	Clean Data (Gb)	Average Read Length of Raw Reads (bp)	Sequencing Coverage (X)
Illumina	250	105.23	105.01	150	148.54
PacBio	20,000	80.61	—	8,530.75	113.78
Hi-C	—	119.15	58.97	150	168.18
Total	—	304.99	—	—	430.50

Note: The genome size of *L*. *crocea* used to calculate sequencing coverage was 708.47 Mbp, which was estimated using a K-mer analysis of the short reads.

HWM gDNA was used in DNA template preparation for sequencing on the PacBio System following the “Template Preparation and Sequencing Guide” provided by Pacific Biosciences (Menlo Park, CA, USA). The main steps were as follows: extracted DNA was first sheared into large fragments (10 Kbp on average) and then purified and concentrated using AMPure PB beads; DNA damage and ends induced in the shearing step were repaired; blunt hairpins were subsequently ligated to the repaired fragment ends; prior to sequencing, the primer was annealed to the SMRTbell template, and then, DNA polymerase was bound to the annealed templates; finally, DNA sequencing polymerases were bound to the primer-annealed SMRTbell templates.

After sequencing, a total of 9.45 K (80.61 Gbases) long reads were generated from the PacBio SEQUEL platform. The average length and N50 length of these reads were 8,530.75 bp and 12,624 bp, respectively. The genome size of *L*. *crocea* was estimated to be 708.47 Mbp using K-mer analysis, and the average sequencing coverage was estimated as 113.78X (Table 1).

Hi-C sequencing was performed parallel to the PacBio sequencing. We used formaldehyde to fix the conformation of the HMW gDNA. Then, the fixed DNA was sheared with MboI restriction enzyme. The 5′ overhangs induced in the shearing step were repaired using biotinylated residues. Following the ligation of blunt-end fragments *in situ*, the isolated DNA was reverse-crosslinked, purified, and filtered to remove biotin-containing fragments. Subsequently, DNA fragment end repair, adaptor ligation, and polymerase chain reaction (PCR) were performed successively. In the end, sequencing was performed on the Illumina HiSeq2500 platform and yielded a total of 119.15 Gb paired-end reads, with an average sequencing coverage of 168.18X (Table 1).

### *De novo* assembly of the *L*. *crocea* genome

**Fig. 1:**
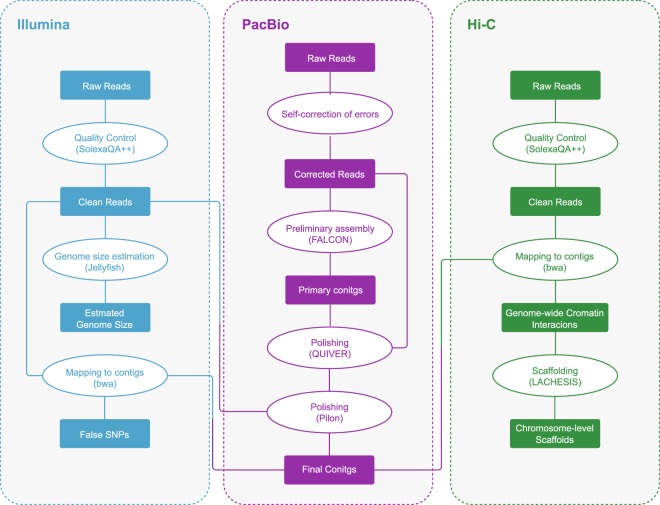
Illustration of the complete genome assembly pipeline.

In summary, as shown in Fig. 1, reads generated from three different types of libraries were used in three different assembly stages separately: Illumina sequencing data were used in estimation of genome size and polishing of preliminary contigs; PacBio sequencing data were used for preliminary contig assembly; and Hi-C reads were used in chromosome-level scaffolding.

The read pairs generated from the small-insert genomic DNA libraries were filtered out if the proportion of “N” sites exceeded 10%, number of low-quality bases exceeded 75 or the reads were polluted by adaptor sequences. Then, all clean Illumina reads were used to generate 17-mers with a window-sliding-like method. Accordingly, there were 4^17^ different 17-mers. After calculating the depth distribution of these 17-mers using Jellyfish^11^ (v2.1.3), we could estimate the genome size using Lander/Waterman’s equations:

1 $${C}_{base}={C}_{17 \mbox{-} mer}\times L/(L \mbox{-} 17+1)$$

2 $${G}_{est}={N}_{17 \mbox{-} mer}/{C}_{17 \mbox{-} mer}={N}_{base}/{C}_{base}$$

In these equations, L is read length (150 for Illumina reads), N_base_ and N_17-mer_ are counts of bases and 17-mers respectively; C_base_ and C_k-mer_ are expected coverage depths of bases and 17-mers, respectively; estimated genome size is represented by G_est_. As a result, the genome size of *L*. *crocea* was estimated to be approximately 708.47 Mbp.

Long reads generated from the PacBio SEQUEL platform containing adaptor sequences or with a quality value lower than 20 (corresponding to a 1% error rate) were filtered out. The remaining reads were subsequently further processed by self-correction to address sequencing errors using Falcon^12^ (version 1.8.2). Thereafter, genome assembly based on these error-corrected reads was processed in three stages: detection of overlaps among input reads and assemble the final string graph^13^ using the Falcon pipeline; calling of highly accurate consensus sequences based on PacBio reads using quiver^14^ (version 2.1.0); and polishing the preliminary contigs with Illumina reads using pilon^15^ (version 1.21). Finally, we obtained a newly assembled genome of *L*. *crocea* containing 1,591 contigs with a total length of 723.86 Mb and a contig N50 length of 2.83 Mb (Table 2).

**Table 2:** Summary of the *L*. *crocea* genome assembly and structural annotation.

**Genome Assembly**	
Contig N50 length (Mbp)	2.83
Number of conitgs longer than N50	68
Contig N90 size (Kbp)	0.26
Number of conitgs longer than N90	376
Number of conitgs	1,591
Maximum contig length (Mbp)	11.8
Median contig length (Mbp)	0.64
Total contig length (Mbp)	723.86
**Structural Annotation**	
Number of protein-coding genes	23,172
Number of unannotated genes	73
Average transcript length (bp)	11,839.98
Average exons per gene	9.27
Average exon length (bp)	158.16
Average CDS length (bp)	1,465.51
Average intron length (bp)	1,255.04

To obtain chromosome-level scaffolds, Hi-C reads were filtered in the same way as we filtered the short-insert library reads and subsequently mapped to *de novo* assembled contigs to construct contacts among the contigs using bwa^16^ (version 0.7.17) with the default parameters. BAM files containing Hi-C linking messages were processed by another round of filtering, in which reads were removed if they were not mapped to the reference genome within 500 bp from the nearest restriction enzyme site. Then, LACHESIS^17^ (version 2e27abb) was used for ultra-long-range scaffolding of *de novo* genome assemblies using the signal of genomic proximity provided by the Hi-C data. In this step, all parameters were set to defaults except that CLUSTER_N, CLUSTER_MIN_RE_SITES and ORDER_MIN_N_RES_IN_SHREDS were set to 24, 80 and 10, respectively. The parameter CLUSTER_N was used to specify the number of chromosomes. For large yellow croaker, this number was determined to be 24 in previous studies^5,18,19^. Ultimately, we obtained 24 chromosome-level scaffolds constructed from 548 contigs with a total length of 668.67 Mb (92.48% of the total length of all contigs) (Table 3).

**Table 3:** Detailed results of chromosome-level scaffolding using Hi-C technology.

Chromosomes	Length (Mbp)	Number of Contigs
Chr1	34.89	34
Chr2	24.81	19
Chr3	28.07	17
Chr4	29.96	22
Chr5	33.77	25
Chr6	24.87	16
Chr7	31.52	27
Chr8	32.80	24
Chr9	24.26	18
Chr10	27.49	16
Chr11	34.65	24
Chr12	26.70	25
Chr13	16.24	24
Chr14	29.81	21
Chr15	27.79	19
Chr16	20.01	23
Chr17	25.06	18
Chr18	32.81	20
Chr19	29.92	30
Chr20	32.24	39
Chr21	27.85	20
Chr22	27.44	11
Chr23	23.57	27
Chr24	22.13	29
Linked Total	668.67	548
Unlinked Total	54.39	1,043
Linked Percent	92.48	34.44
Total	723.06	1,591.00

### Gene annotation

**Fig. 2:**
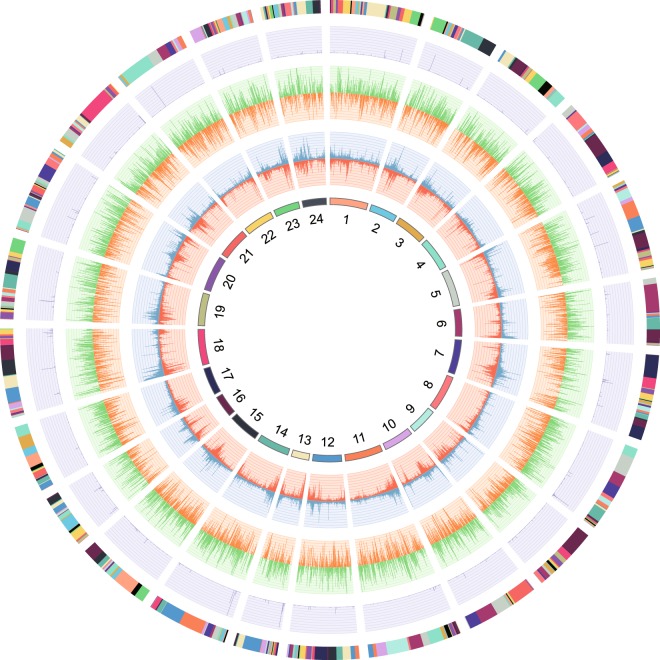
Circos plot of 24 chromosome-level scaffolds, representing annotation results of genes, ncRNAs and transposable elements on these scaffolds. The tracks from inside to outside are: 24 chromosome-level scaffolds, gene abundance of positive strand (red), gene abundance of negative strand (blue), TE abundance of positive strand (orange), TE abundance of negative strand (green), ncRNA abundance of both strands, and contigs that comprised the scaffolds (adjacent contigs on a scaffold are shown in different colours).

To obtain a fully annotated *L*. *crocea* genome, three different approaches were employed to predict protein-coding genes. *Ab intio* gene prediction was performed on the repeat-masked *L*. *crocea* genome assembly using Augustus^20^ (version 2.5.5), GlimmerHMM^21^ (version 3.0.1), Geneid^22^ (version 1.4.4) and GenScan^23^ (version 1.0). Furthermore, homology-based prediction was performed using protein sequences of three common model species [*Danio rerio* (Dre)^24^, *Homo sapiens* (Hsa)^25^, and *Mus musculus* (Mmu)^26^] downloaded from European Nucleotide Archive (ENA) and two related species [*Oreochromis niloticus* (Oni)^27^ and *Notothenia coriiceps* (Nco)^28^]. Subsequently, these protein sequences were mapped onto the generated assembly using blat^29^ (version 35) with a cut off of e-value ≤ 1e^−5^. GeneWise^30^ (version 2.2.0) was employed to align the homologs in the *L*. *crocea* genome against the other species for gene structure prediction. In addition, we also applied transcriptome-based prediction by using existing RNA-seq data generated from various tissues including gonad^31^, spleen^32^, liver^33^, muscle^34^, skin^35^, brain^36^ and embryos in different developmental stages^37^ (Table 4). The RNA-seq reads were mapped onto the genome assembly using TopHat^38^ (version 2.0.13), and the structures of all transcribed genes were predicted by Cufflinks^39^ (version 2.2.1) with the default parameters. The predicted gene sets generated from these three approaches were then integrated to produce a non-redundant gene set using EvidenceModeler^40^ (version 1.1.0). PASA^41^ (version 2.0.2) was then used to annotate the gene structures. As a result, a total of 23,172 protein-coding genes were predicted and subsequently annotated. The average number of exons per gene, and average CDS length were 9,27 and 1465.51 bp, respectively. To identify candidate non-coding RNA (ncRNA) genes, we aligned genome sequences against the Rfam database^42^ (version 12.0) using BLASTN to search for homologs. As a result, a total of 7262 ncRNA genes were predicted (1246 miRNAs, 3517 tRNAs, 1758 rRNAs and 741 snRNAs, Fig. 2 and Table 5).

**Table 4:** List of RNA-seq datasets used for gene structural prediction.

Run	Tissue	Sample Name	Study	BioProject	MBases	Load Date
SRR6474596	gonad	Male5	SRP128079	PRJNA368644	3,824	2018/1/15
SRR6474594	gonad	Female3	SRP128079	PRJNA368644	4,845	2018/1/15
SRR6474588	gonad	Female5	SRP128079	PRJNA368644	4,052	2018/1/15
SRR6474586	gonad	Male4	SRP128079	PRJNA368644	3,742	2018/1/15
SRR5121288	embryo	pharyngula	SRP095312	PRJNA357970	4,399	2016/12/23
SRR5121287	embryo	gastrulation	SRP095312	PRJNA357970	4,392	2016/12/23
SRR5121286	embryo	1_cell_embryo	SRP095312	PRJNA357970	4,567	2016/12/23
SRR5121204	embryo	blastula_L1	SRP095312	PRJNA357970	4,695	2016/12/23
SRR5121203	embryo	256_cell_embryo_L1	SRP095312	PRJNA357970	4,730	2016/12/23
SRR5121202	embryo	16_cell_embryo_L1	SRP095312	PRJNA357970	4,688	2016/12/23
SRR5121194	embryo	8_cell_embryo_L1	SRP095312	PRJNA357970	4,425	2016/12/23
SRR5121193	embryo	2_cell_embryo_L1	SRP095312	PRJNA357970	4,495	2016/12/23
SRR5000825	spleen	BS24h	SRP092778	PRJNA340054	5,229	2016/11/7
SRR5000824	spleen	BS0h	SRP092778	PRJNA340054	5,278	2016/11/7
SRR3711298	liver	The raw sequence reads of *Larimichthys crocea* liver	SRP076957	PRJNA326556	4,758	2016/6/27
SRR3711297	liver	The raw sequence reads of *Larimichthys crocea* liver	SRP076957	PRJNA326556	4,878	2016/6/27
SRR2984347	skin	stress_0.5h_1	SRP066525	PRJNA303096	2,963	2015/12/11
SRR2984346	skin	control	SRP066525	PRJNA303096	2,913	2015/12/11
SRR2473991	muscle	GSM1890206	SRP063956	PRJNA296537	5,073	2015/9/21
SRR2473990	muscle	GSM1890205	SRP063956	PRJNA296537	6,310	2015/9/21
SRR1509885	mixture	a composite sample of large yellow croaker	SRP044199	PRJNA254539	6,122	2014/7/10
SRR1284627	brain	GSM1385502	SRP041934	PRJNA246784	6,144	2015/12/29
SRR1284623	brain	GSM1385498	SRP041934	PRJNA246784	4,399	2015/9/13

**Table 5:** Detailed results of ncRNA annotation.

Type		Copy	Average Length (bp)	Total Length (bp)	Proportion in Genome (‰)
miRNA		1,246	100.90	125,725	0.17
tRNA		3,517	75.58	265,811	0.37
rRNA	18S	68	227.37	15,461	0.02
	28S	70	208.07	14,565	0.02
	5.8S	1	45	45	0.00
	5S	1,619	111.3	180,190	0.25
	Subtotal	1,758	119.6	210,261	0.29
snRNA	CD-box	153	118.72	18,164	0.03
	HACA-box	119	156.36	18,607	0.03
	Splicing	469	124.25	58,271	0.08
	Subtotal	741	129.85	95,042	0.14
Total		72	95.96	696,839	0.97

Note: The genome size of *L*. *crocea* was estimated to be 708.47 Mbp by genome K-mer analysis.

Gene function annotations were conducted against the NCBI nr and SwissProt protein databases, and homologs were called with E values of <1 × 10^−5^. The functional classification of Gene Ontology (GO) categories was performed using the InterProScan program^43^ (version 5.26). Kyoto Encyclopedia of Genes and Genomes (KEGG)^44^ pathway annotation analysis was performed using the KEGG Automatic Annotation Server (KAAS)^45^. As a result, a total of 23,323 genes could be annotated, accounting for 99.7% of all predicted genes (Fig. 2, and Table 2).

### Repetitive element characterization

**Fig. 3:**
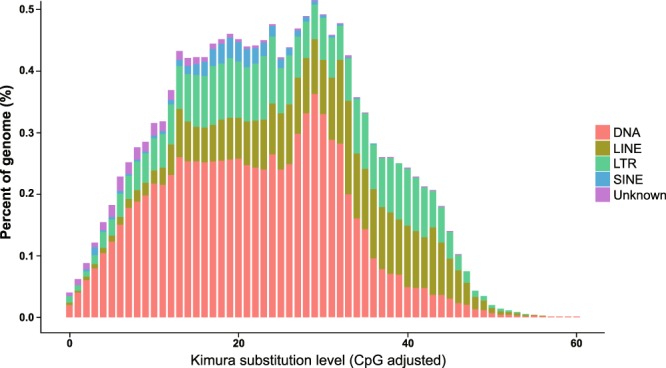
Divergence distribution of TEs in the *L*. *crocea* genome.

We employed two approaches to detect repeat sequences in the *L*. *crocea* genome. First, we used Tandem Repeats Finder^46^ (version 4.04), Piler^47^ (version 1.0), LTR_FINDER^48^ (version 1.0.2), RepeatModeler^49^ (version 1.04) and RepeatScout^50^ (version 1.0.2) to detect various kinds of repeat sequences in the *L*. *crocea* genome synchronously. The results were then integrated as a *de novo* non-redundant repeat sequence library by USEARCH^51^ (version 10.0.240). Subsequently, the library was annotated using RepeatMasker^49^ (version 3.2.9) based on Repbase TE^52^ (version 14.04) to discriminate between known and novel transposable elements (TEs). In another approach, genome sequences were mapped on Repbase TE^52^ (version 14.04) using RepeatProteinMask^49^ (version 3.2.2), a Perl script included in RepeatMasker, to detect transposable element (TE) proteins in *L*. *crocea* genome. After combining the results of the two approaches and removing the redundancy, ~26.13% of the *L*. *crocea* genome with a total length of 189.3 Mb were identified as repetitive elements, including 69.1 Mb (9.54%) of DNA transposons, 51.4 Mb (7.09%) of long interspersed nuclear elements (LINEs) and 52.4 (7.24%) of long terminal repeats (LTRs) (Table 6). A Perl script createRepeatLandscape.pl supplied with RepeatMasker was used to visualize the divergence distribution of TEs in the *L*. *crocea* genome (Fig. 3). The numbers and lengths of contigs comprising each chromosome were shown in the outermost track of a Circos^53^ plot.

**Table 6:** Detailed classification of repeat sequences.

Type	*De novo*		TE proteins		Combined TEs	
	Length (Mbp)	Proportion in Genome (%)	Length (Mbp)	Proportion in Genome (%)	Length (Mbp)	Proportion in Genome (%)
DNA	66.39	9.17	5.58	0.77	69.11	9.54
LINE	45.38	6.26	14.50	2.00	51.37	7.09
SINE	3.45	0.48	0.00	0.00	3.45	0.48
LTR	51.19	7.07	9.51	1.31	52.41	7.24
Simple Repeat	16.86	2.33	0.00	0.00	16.86	2.33
Unknown	11.85	1.64	0.00	0.00	11.85	1.64
Total	183.50	25.33	29.51	4.07	189.27	26.13

Note: “*De novo*” represents the *de novo* identified transposable elements using RepeatMasker, RepeatModeler, RepeatScout, and LTR_FINDER. “TE proteins” indicates homologous transposable elements in Repbase identified with RepeatProteinMask, while “Combined TEs” refers to the combined results of transposable elements identified in these two ways. “Unknown” represents transposable elements that could not be classified by RepeatMasker.

## Data Records

This whole genome shotgun sequencing project has been deposited at DDBJ/ENA/GenBank under the accession RQIN00000000. The version described in this paper is version RQIN01000000^54^.

Genome assembly and annotation have also been deposited at Figshare^55^.

All sequencing data, including the PacBio long reads, Illumina short reads and Hi-C reads, have been deposited in the NCBI Sequence Read Archive (SRA) under the accession numbers SRP169057^56^.

The existing RNA-seq datasets are all available in NCBI SRA, with the accession numbers listed in Table 4 ^31–37^.

## Technical Validation

### DNA sample quality

DNA quality was assessed using 1% agarose gel.

### Illumina libraries

Ready-to-sequence Illumina libraries were quantified by qPCR using the KAPA Library Quantification Kit for Illumina Libraries (KapaBiosystems, Wilmington, MA, USA), and library profiles were evaluated with an Agilent 2100 Bioanalyzer (Agilent Technologies, Santa Clara, CA, USA).

### Completeness and accuracy of the assembly

The completeness and accuracy of the assembly were further assessed in multiple ways. First, the reads from the short-insert library were re-mapped onto the assembly using bwa^16^ (version 0.7.17). As a result, 97.61% of the reads were accurately mapped with a coverage of 99.89%. Then Genome Analysis Toolkit^57^ (GATK) (version 4.0.2.1) was applied for SNP discovery and finally identified 3,739.45 K SNPs, including 3,735.88 K heterozygous SNPs and 3568 homozygous SNPs (Table 7). The extremely low proportion of homozygous SNPs suggests the high accuracy of this assembly. The assembly completeness was evaluated using Core Eukaryotic Genes Mapping Approach (CEGMA) software^58^ (version 2.3) based on an appropriate reference gene set, core vertebrate genes (CVG)^59^. There were 232 genes out of the complete set of 233 genes (99.57%) covered by the assembly, suggesting the high completeness of the draft genome of *L*. *crocea* (Table 7). Subsequently, Benchmarking Universal Single-Copy Orthologs (BUSCO) software^60^ (version 1.22) was executed using actinopterygii_odb9 database to assess the predicted gene set. The genome mode result showed that 97.1% of all 4584 BUSCOs were assembled, including 93.7% and 3.3% of all BUSCOs were completely and partially assembled, also implying a high level of completeness for the *de novo* assembly (Table 7). In addition, the results generated with protein mode based on all predicted genes showed that 91.2% of all 4584 BUSCOs were assembled, including 11.9% of all BUSCOs that were partially predicted (Table 7).

**Table 7:** Details of accuracy and completeness validation of genome assembly.

**Illumina Reads Mapping**		
Mapping ratio	97.61%	
Mapping coverage	99.89%	
Number of heterozygous SNPs	3,735,880	
Number of homozygous SNPs	3568	
**CEGMA**		
Total number of reference genes	233	
Number of completely assembled CEGs	231	
Proportion of completely assembled CEGs (%)	99.14	
Number of assembled CEGs	232	
Proportion of assembled CEGs (%)	99.57	
**BUSCO (genome mode)**	**Number**	**Proportion (%)**
All orthologues used	4584	100.00
Complete and fragmented orthologues	4419	97.1
Missing orthologues	135	2.9
**BUSCO (protein mode)**	**Number**	**Proportion (%)**
All orthologues used	4584	100.00
Complete and fragmented orthologues	4182	91.2%
Missing orthologues	402	8.8

## Data Availability

The execution of this work involved using many software tools. To allow readers to repeat any steps involved in genome assembly and genome annotation, the settings and parameters were provided below: Genome assembly: **(1) Falcon**: all parameters were set to the defaults; (**2) quiver**: all parameters were set to the defaults; (**3) pilon**: all parameters were set to the defaults; (**4) LACHESIS**: RE_SITE_SEQ = AAGCTT, USE_REFERENCE = 0, DO_CLUSTERING = 1, DO_ORDERING = 1, DO_REPORTING = 1, CLUSTER_N = 24, CLUSTER_MIN_RE_SITES = 300, CLUSTER_MAX_LINK_DENSITY = 4, CLUSTER_NONINFORMATIVE_RATIO = 10, REPORT_EXCLUDED_GROUPS = −1; Genome annotation: **(1) RepeatProteinMask**: -noLowSimple -pvalue 0.0001 -engine wublast; (**2) RepeatMasker**: -a -nolow -no_is -norna -parallel 1; (**3) LTR_FINDER**: -C -w 2; (**4) RepeatModeler**: -database genome -engine ncbi -pa 15; (**5) RepeatScout**: all parameters were set to the defaults; (**6) TRF**: matching weight = 2, mismatching penalty = 7, INDEL penalty = 7, match probability = 80, INDEL probability = 10, minimum alignment score to report = 50, maximum period size to report = 2000, -d –h; (**7) Augustus**:–extrinsicCfgFile–uniqueGeneId = true–noInFrameStop = true–gff3 = on–genemodel = complete–strand = both; (**8) GlimmerHMM**: -f –g; (**9) Genscan**: -cds; (**10) Geneid**: -P -v -G -p geneid; (**11) Genewise**: -trev -genesf -gff –sum; (**12) BLAST**: -p tblastn -e 1e-05 -F T -m 8 -d; (1**3) EVidenceModeler**: G genome.fa -g denovo.gff3 –w weight_file -e transcript.gff3 -p protein.gff3–min_intron_length 20 (**14) PASA**: all parameters were set to the defaults.
